# Smoothing splines of apex predator movement: Functional modeling strategies for exploring animal behavior and social interactions

**DOI:** 10.1002/ece3.8294

**Published:** 2021-12-09

**Authors:** Andrew B. Whetten

**Affiliations:** ^1^ Department of Mathematical Sciences University of Wisconsin – Milwaukee Milwaukee Wisconsin USA

**Keywords:** animal behavior, animal interaction, animal telemetry, apex predators, functional data analysis, information theory, jaguar, mutual information, smoothing splines

## Abstract

The collection of animal position data via GPS tracking devices has increased in quality and usage in recent years. Animal position and movement, although measured discretely, follows the same principles of kinematic motion, and as such, the process is inherently continuous and differentiable. I demonstrate the functionality and visual elegance of smoothing spline models. I discuss the challenges and benefits of implementing such an approach, and I provide an analysis of movement and social interaction of seven jaguars inhabiting the Taiamã Ecological Station, Pantanal, Brazil, a region with the highest known density of jaguars. In the analysis, I derive measures for pairwise distance, cooccurrence, and spatiotemporal association between jaguars, borrowing ideas from density estimation and information theory. These measures are feasible as a result of spline model estimation, and they provide a critical tool for a deeper investigation of cooccurrence duration, frequency, and localized spatio‐temporal relationships between animals. In this work, I characterize a variety of interactive relationships between pairs of jaguars, and I particularly emphasize the relationships in movement of two male–female and two male–male jaguar pairs exhibiting highly associative relationships.

## Graphical abstract

I propose the use of smoothing spline models to explore animal movement and social interaction. I propose temporal‐state density estimation and localized mutual information measures to characterize behavior and strength of association between jaguars on a refined and unified time grid. I provide further characterization of time‐dependent male–male and female–male relationships in this region, an important step in determining conservation policy for an endangered neotropical predator.

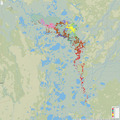

## 1 INTRODUCTION

Technological advancements in remote sensing of animal movement, referred to as animal telemetry, have revolutionized the discipline of movement ecology. Animal movement data provides critical information about ecological processes, and it can be a vital asset to conservation efforts of species and ecosystems. The increased feasibility of tracking and collecting animal movement information has yielded large reservoirs of fine‐scale spatio‐temporal data, and the challenges of meaningfully modeling animal behavior have resulted in the expansion of holistic machine learning methodology that appropriately considers animal psychology and cognition (Buderman et al., 2016; Hooten et al., 2017; Lewis et al., 2021).

The analysis of animal telemetry data has a number of challenges. (1) Spatial and temporal density of measurements is subject to extreme variation. Animal behaviors may shift phenologically between migratory and residency states, and even for nonmigratory species, this problem can present itself in a smaller scale region as animals shift between resting, foraging, or transit states. Temporal density variation may be caused by loss of connection, malfunctioning, and damage of the device over time. (2) Even with advancements in precision and reliability of animal tracking, the datasets are inherently discrete, and any analysis of such data requires a conscious choice between modeling such processes discretely or attempting to model them continuously. (3) Animal behavior cannot be univariately characterized. Animal movement is characterized by position, rate of change of position, and cooccurrence with other animals, all of which may suddenly shift under interactions with an array of environmental factors that alter the allocation of critical resources for survival (Hooten et al., 2017; Lewis et al., 2021).

Discrete time methods have had steady use in the field (Jonsen et al., 2005; McClintock et al., 2014, 2015; Morales et al., 2004), but recent literature has provided significant progress in continuous‐time modeling (Hanks et al., 2015; Harris & Blackwell, 2013; Johnson et al., 2008; Parton & Blackwell, 2017). Animal movement is explicitly continuous, like any kinematic process, and continuous‐time models celebrate and take advantage of this continuity in the modeling process. These models are exceptional and flexible tools for modeling the complexity of animal movement. However, I emphasize that we should more fully embrace animal movement as a kinematic process. We must acknowledge that projectile movement in a real space $R^{2}$ is smooth, and I propose that we further consider modeling strategies and methodological developments that account for the 1st and 2nd‐order differentiation of an animal movement processes.

I present a philosophically different approach for analyzing animal telemetry in which the unit of analysis is a curve (or function) as opposed to single site measurements. This approach widely referred to as functional data analysis (FDA) roots in the assumption that measurements vary over some continuum such as space or time, and that there is an underlying smoothness inherent to the process of interest (Ramsay & Dalzell, 1991; Ramsay & Silverman, 2005; Ullah & Finch, 2013). The assembly of an entire smooth curve of an animal's movement is accomplished using linear combinations basis functions, which are the foundation of smoothing spline models. They are widely acclaimed for their ability to model complex and noisy data (Ramsay & Silverman, 2005).

Animal movement is a visual spectacle, and the statistical visualization of animal movement is greatly aided using smoothing splines. FDA methods provide a viable and accessible option for examining an estimated complete path and the speed and acceleration (and deceleration) along this path, which are vital in the classification of various types of animal behavior. There have been recent basis function models proposed to model animal movement (Anderson‐Sprecher & Lenth, 1996; Buderman et al., 2016; Hefley et al., 2017; Henning et al., 2017; Hooten & Johnson, 2016), but there is great need to incorporate a wide array of strategies for an appropriate and application‐specific exploration using smoothed spline models.

In this project, I analyze and visualize the movement of seven Jaguars inhabiting the Taiamã Ecological Station, Pantanal, Brazil, and the associative and cooccurrence relationships between them. Fine‐scale movement of jaguars in this region has recently been explored using association rule mining algorithms to study their behavior and social interaction. Identifying behavioral changes and social interactions are crucial aspects of species ecology, and this recent work has added to literature of jaguar territory sharing (Fontes et al., 2021). Jaguars are generally solitary and territorial apex predators, but in areas with high primary productivity, the overlap of territory and its effects on mating, cooperation, and competition yield a complex system of interdependent subjects that can directly or passively interact (Cavalcanti & Gese, 2009; Fontes et al., 2021; Morato et al., 2016).

I construct smoothing spline models to continuously and differentiably characterize the movement, resting, and migratory behavior of these 7 jaguars. These smoothing spline models provide exceptional fit, and they provide the means to feasibly measure animal association using a measure of mutual information from the discipline of information theory. Further, I introduce the concept of a *Cooccurrence Potential Plots*, which are smooth density functions derived from the distance between pairs of jaguars on the refined and unified grid. The refinement and unification of the time‐grid is an inherent and advantageous by‐product of spline models.

In efforts to improve upon the previous work in Fontes et al., this analysis accomplishes two primary objectives: (1) An estimation of cooccurrence potential, which has a conservative theoretical standing in the presence of measurement error for lower raw time resolution and allows for inferences to be made between observations on the raw time grid, and (2) a derivation of a correlation function based on animal movement, which captures shifts in the associations between individuals. In (1), the conservative theoretical nature of this measure refers to the difference between cooccurrence potential and cooccurrence frequency, where cooccurrence frequency is a count of the amount of times that two animals occur within a certain spatial and temporal radius, and cooccurrence potential is a measure of the density of time values on a time grid where two animals are within a radius where there is high probability of cooccurrence based on the ability of the animals to interact in between time observations. The use of a density measure, such as cooccurrence potential, can be more conservative since the constructed probability density function identifies time periods where interaction is more likely as opposed to a simple count of time points. This application of FDA methods to animal movement showcases the plausibility of studying animal movement with the theoretical backing of the laws of kinematic motion, and most importantly, the approach provides an increased set of tools to improve the study animal movement in relation to dynamic social and environmental factors.

In this project, it is important to acknowledge that measurement error is not considered since this attribute was not recorded in the public version of the data product. The exact specifications of the utilized GPS tracking devices and a disclaimer regarding the data quality are detailed in the following section. FDA methods exists to address measurement error for various disciplines (Buderman et al., 2016; Cai, 2015; Sneha & Ma, 2020), and I leave this important and interesting aspect of animal telemetry to future work.

## 2 METHODS

Fitting smoothed spline models provides a number of advantages for irregularly and sparsely measured data that is known to vary over some continuum, but it is important to note that some sacrifice of position is made in a model that aims to smooth a function through a series of measurements (Ramsay & Silverman, 2005). More specifically, smoothing spline models differ from interpolation models since the objective of interpolation is to fit a function that crosses through all recorded measurements of a process with an error of zero, where as for smoothing splines, the objective is to fit a simpler function that captures the main features of the process while minimizes the error between the optimal function and the recorded measurements. Generally, smoothing splines are more informative as they prevent over‐fitting to noise in the raw data, which can obscure critical features of a process. Since GPS positioning systems have known measurement error (even though measurement error is not reported in this data product), I aim to show that this sacrifice is worth the benefits of this approach, and further that modifications to the model can be instated to adapt and improve this approach.

### 2.1 Fitting smoothed spline models to jaguar movement

For a collection of raw hourly recordings of a single jaguar's position, denoted by $Y_{\text{lat}}=\left[y_{\text{lat}1}\ldots y_{\text{lat}n}\right]$ and $Y_{\text{lon}}=\left[y_{\text{lon}1}\ldots y_{\text{lon}n}\right]$, I estimate ${\hat{x}}_{\text{lat}}\left(t\right)=\sum_{k=1}^{K}c_{\text{lat}k}\phi_{k}\left(t\right)$ and ${\hat{x}}_{\text{lon}}\left(t\right)=\sum_{k=1}^{K}c_{\text{lon}k}\phi_{k}\left(t\right)$ subject to a roughness penalty on the second derivative of the basis expansion $\Phi =\left[\phi_{1}\left(t\right)\ldots \phi_{K}\left(t\right)\right]$ where $c_{k}$ are the coefficients of the terms of the basis expansion denoted by $\phi_{k}$, which in this project is constructed using a B‐spline basis expansion (De Boor, 1978; Ramsay & Silverman, 2005). Both latitude and longitudinal movement can be individually expressed as an unconstrained minimization defined by

(1) $$\min_{\vec{c}}\|\vec{y}‐\Phi \vec{c}{\|}^{2}+\lambda c^{T}Rc\;\text{for}\;\lambda \geq 0,$$

where $R_{\mathrm{jk}}=\sum_{l=1}^{M}\phi_{j}^{\prime \prime }\left({\tilde{t}}_{l}\right)\phi_{k}^{\prime \prime }\left({\tilde{t}}_{l}\right)h\;\text{for}\;h={\tilde{t}}_{l}‐{\tilde{t}}_{l‐1}$ (Ramsay & Silverman, 2005). We select an appropriate value for *λ* using the optimal lambda for a single site determined by the generalized cross‐validation criteria,

(2) $$\text{GCV}=\frac{\text{MSE}(\lambda )}{\left(1‐\frac{df_{\lambda }}{M}\right)}.$$

For jaguar movement, I have fitted the spline models with low or negligible roughness penalization, since the precision of movement is of high priority. The roughness of the movement can also be restricted by latitude and longitude separately, which may be on interest if we seek to model movement with substantial differences in between latitudinal and longitudinal behaviors (such as long distance ungulate or bird migration), but for this work the roughness is penalized equally for both dimensions. The resulting smoothed jaguar movement curves have the form

(3) $$\begin{array}{c} {\hat{x}}_{\text{lat}}=\Phi {\left(\Phi^{T}\Phi +\lambda_{\text{lat}}R\right)}^{‐1}\Phi^{T}\vec{y}=S_{\text{lat}}{\vec{y}}_{\text{lat}},\\ {\hat{x}}_{\text{lon}}=\Phi {\left(\Phi^{T}\Phi +\lambda_{\text{lon}}R\right)}^{‐1}\Phi^{T}\vec{y}=S_{\text{lon}}{\vec{y}}_{\text{lon}}. \end{array}$$

The jaguar's 2‐dimensional movement is then characterized by coordinates on the path $\left({\hat{x}}_{\text{lon}}\left(t\right),{\hat{x}}_{\text{lat}}\left(t\right)\right)$, which has been done similarly in recent work (Buderman et al., 2016; Hooten et al., 2018). I note that Equations (2) and (3) jointly characterize a two‐parameter search for $\lambda_{\text{lat}}$ and $\lambda_{\text{lon}}$. In all cases, although an optimal GCV criterion can be detected, some additional tuning by visual inspection was performed, and this is a common practice when constructing spline models to ensure that critical shifts in animal position are being correctly captured by the model. With a GPS tracking device of sufficient resolution, critical shifts in behavior should be discernible in the presence of measurement error, and because of this it is important to not rely solely on an optimization criteria when fitting such a model. Over‐fitting permits too much roughness in the model which ascribes measurement error to ecological behavior, and under‐fitting ascribes actual movement to measurement error.

In order to meaningfully estimate jaguar position across highly disparate densities of raw time recordings, careful placement of knots is advised. Let $\left(t_{1},\ldots ,t_{n}\right)$ be independently and identically distributed time samples from an unknown distribution $f_{h}$. We estimate the density of sampled times for a given jaguar using kernel density estimation defined by ${\hat{f}}_{h}\left(t\right)=\left(1/nh\right)\sum_{i=1}^{n}K\left((t‐t_{i})/h\right)$, where *K* is gaussian kernel function and *h* is a smoothing bandwidth parameter where higher values of *h* yield a smooth estimate of the density (Wand & Jones, 1995). Let $k={\hat{f}}_{h}\left(t^{*}\right)$ be selected as a threshold where $t_{i}$ with $\hat{f}\left(t_{i}\right)>k$ define the collection of high density times $\left\{t_{i}|f_{h}\left(t_{i}\right)\rangle k\right\}=\left(\tau_{1},\ldots ,\tau_{m}\right)$ where $\tau_{1}<\ldots <\tau_{m}$. This selection of knots is carefully placed to avoid over fitting regions of the time domains that are barren or extremely sparse. This is desirable for periods where GPS tracking devices are out‐of‐operation for an extended period, but it is still desirable to fit regions with dense recordings with high precision.

Continuous‐time estimation of distance and speed has been developed for standard continuous time models (Noonan et al., 2019). In the next two sections, I outline a derivation of speed and distance measures for animal movement in the FDA paradigm.

### 2.2 Differentiation of the smoothed position functions and derivation of rest period density functions

Differentiation of the smoothed position paths is then conveniently estimated using the same collection of coefficients, ${\vec{c}}_{\text{lat}}$ and ${\vec{c}}_{\text{lon}}$, and the derivation functions are defined by

(4) $${\hat{x}}_{\text{lat}}^{\prime }\left(t\right)=\sum_{k=1}^{K}c_{\text{lat}k}\phi_{k}^{\prime }\left(t\right),\;{\hat{x}}_{\text{lon}}^{\prime }\left(t\right)=\sum_{k=1}^{K}c_{\text{lon}\;k}\phi_{k}^{\prime }\left(t\right)$$

where $\phi_{k}^{\prime }\left(t\right)$ is the derivative of the basis expansion (Buderman et al., 2016, 2018; Ramsay & Silverman, 2005). The estimated speed of jaguar position can then be defined by

(5) $${\hat{x}}^{\prime }\left(t\right)=\sqrt{{\left({\hat{x}}_{\text{lat}}^{\prime }\left(t\right)\right)}^{2}+{\left({\hat{x}}_{\text{lon}}^{\prime }\left(t\right)\right)}^{2}}.$$

Behavioral states of animal movement are generally characterized by different speed of movement. As an example, a resting state should be characterized by lower estimated speeds, while migratory, foraging, and other transitory states are characterized by faster speeds. For this project, I used a speed of 0.25 m/s as a cutoff between resting and transit states. Clearly, a literal resting state should have a derivative value of zero, so in this application resting state has a looser interpretation that characterized by stationary and exceptionally small changes in position. Similar to before, I subset “resting state” times and derive a kernel density function for the distribution of resting times,

(6) $${\hat{f}}_{h}\left(t\right)=\frac{1}{\mathrm{nh}}\sum_{i=1}^{n}K\left(\frac{t‐t_{i}}{h}\right),$$

where *K* is Gaussian kernel function and *h* is a smoothing bandwidth parameter where $t_{1},\ldots ,t_{n}$ are restricted to the set $\left\{t_{i}|{\hat{x}}^{\prime }\left(t\right)<1\right\}$.

### 2.3 Pairwise Jaguars distance functions and derivation of cooccurrence potential plots

For any pairs of jaguars, *J*_1_ and *J*_2_, with geographic position monitored on the domain [*a*,*b*] and [*c*,*d*], respectively, with a<c<b<d, a distance measure can be defined between pairwise estimations of position on the refined regular time grid $t_{1},\ldots ,t_{p}$ where $c=t_{1}$ and $b=t_{p}$, and the distance metric in this work is the WGS84 ellipsoidal distance (Hijman, 2019). This regular time grid is subsetted from the refined global time grid used to smooth jaguar position; in this work the refined grid provides an estimate of position every 60 min. Although not finer than the raw grid, this grid resolution was chosen since already provides extensive interpolation of missing hours, and the smoothed spline model are smaller in size. The choice of time grid is arbitrary in the FDA paradigm, and it can be readily refined to a desired resolution. As an example, the smoothed spline models implemented in this project could be refined to provide 1‐minute estimations, and they would still follow the same smoothed path defined on the selected resolution. There may be clear advantages by estimating movement on this resolution, but this is a question that will be left to future work.

Cooccurrence potential in this work is defined as a density function of times from the refined and unified time grid where the distance between a 2 or more jaguars is within a certain threshold. This work only examines pairwise cooccurrence potential, but I discuss the extension to greater than two jaguars in the Discussion Section. More specifically, I define the cooccurrence potential function by

(7) $${\hat{C}}_{h}\left(t\right)=\frac{1}{\mathrm{nh}}\sum_{i=1}^{n}K\left(\frac{t‐t_{i}}{h}\right),$$

where *K* is Gaussian kernel function and *h* is a smoothing bandwidth parameter where $t_{1},\ldots ,t_{n}$ are restricted to the set $\left\{t_{i}|\text{dist}\left({\hat{x}}_{J1}\left(t_{i}\right),{\hat{x}}_{J2}\left(t_{i}\right)\right)<\delta \right\}$. The parameter δ is a distance threshold, and cooccurrence potential for this application is set to δ=1800m. This indicates that times where a pair of jaguars are estimated to be within this threshold have a high probability of (either passive or direct) interaction (Harmsen et al., 2010). This threshold is chosen with the intent to only capture time periods where a high probability of interaction is possible. Higher cooccurrence potential implies that there is a larger volume of times on the refined time grid where a pair of jaguars are in close proximity, indicating that there is the potential for an interaction. In previous work, cooccurrence frequency is defined on the raw time grid for times where a pair of jaguars were within 200–400 m of each other. The raw time grid in this work records positions of jaguars at a maximum of every hour. Within an hour time‐window, it is apparent that jaguars can travel far beyond 200–400 m since an animal walking slowly at 4 km/h in a straight‐line can cover 10 times the distance of 400 m in an hour. A threshold distance of 1800 m is too far to imply direct interaction at a given time; however, there is a probability that two jaguars can interact with each other in between known or estimated positions. For this reason, it is still instructive to compute the density of times where jaguars fall within a larger radius than 400 m.

### 2.4 Mutual information of jaguar movement

Mutual information is a measure of mutual dependence between two random variables, or more simply, the amount of information gained about one variable by observing the other (Cover & Thomas, 1991). Let $\left(X,Y\right)$ be a pair of random variables with values spanning the space $\left(X\times Y\right)$. The mutual information between two jointly continuous random variables *X* and *Y* is defined by

(8) $$I\left(X;Y\right)=\int_{Y}\int_{X}p_{\left(X,Y\right)}\left(x,y\right)\log\frac{p_{\left(X,Y\right)}\left(x,y\right)}{p_{X}\left(x\right)p_{Y}\left(y\right)}dx\;dy$$

where $p_{\left(X,Y\right)}$ is the joint probability density function of *X* and *Y*, and $p_{X}$ and $p_{Y}$ are the respective marginal density functions. It is clear that if *X* and *Y* are independent then information gained from observing one of the random variables does not provide information about the other, and recall that for independent random variables, $p_{\left(X,Y\right)}\left(x,y\right)=p_{X}\left(x\right)p_{Y}\left(y\right)$, which implies from Equation (4) that I(X;Y)=0 (Cover & Thomas, 1991).

To measure dependence or strength of association between pairs of jaguar movements, it is clear that a global measure of mutual information is insufficient to measure correlation between jaguars since their relationships may be dynamic and shifting. I propose the use of the localized mutual information measure $I_{L}$. Other localized mutual information measures have been derived for various applications (Dai et al., 2015; Klein et al., 2008; Owoeye et al., 2018). In this work, $I_{L}$ is defined by

(9) $$I_{L}\left(X;Y\right)=\int_{Y_{L}}\int_{X_{L}}p_{\left(X_{L},Y_{L}\right)}\left(x,y\right)\log\frac{p_{\left(X_{L},Y_{L}\right)}\left(x,y\right)}{p_{X_{L}}\left(x\right)p_{Y_{L}}\left(y\right)}dx\;dy$$

where $X_{L}$ and $Y_{L}$ are restrictions of the random variable to the domain defined by the set $L_{i}=\{t|t\in \left[t_{i}‐\lambda ,t_{i}+\lambda \right]\}$. The parameter *λ* defines the bandwidth or radius over which local mutual information is measured.

Ultimately, the advantage of this approach is to construct a bivariate measure of mutual information, and finally to generate a mutual information function with respect to time. For two bivariate random vectors $X=\left(X_{\text{lat}},X_{\text{lon}}\right)$ and $Y=\left(Y_{\text{lat}},Y_{\text{lon}}\right)$, I define joint local mutual information by

(10) $$I_{L}\left(X,Y|\lambda \right)=\sqrt{I_{L}{\left(X_{\text{lat}};Y_{\text{lat}}\right)}^{2}+I_{L}{\left(X_{\text{lon}};Y_{\text{lon}}\right)}^{2}}.$$

Clearly, various weighting schemes for combining local mutual information for latitude and longitude could be derived. (Also, since the measure of mutual information is measured from the center of an interval, it may be advantageous to weight the contribution of realizations of a random variable in the mutual information computation based on their proximity to the center of the interval although this is not explored here.) Finally, I define the joint local mutual information function with respect to time by

(11) $$I\left(t;\lambda \right)=I_{L_{i}}\left(X,Y|\lambda \right).$$

where $i=1,\ldots ,\dim(\vec{t})$ and $\vec{t}$ is the vector of times from the refined time grid. It is important to note that in this application the values of *t* are limited to the defined resolution of the spline model. So each time $t_{i}$ is associated with a given $L_{i}$, and as such, the pair $\left(t_{i},L_{i}\right)$ defines a centered window $L_{i}$ over which I(t;λ) is evaluated at a given time $t_{i}$.

This derived result can be used to monitor periods of time where high and low correlation between a pair of jaguars is observed, and it provides a tool for monitoring if periodicity in the strength of their relationships exists (Meyer, 2014). An example we might look for would be strength of relationships in movement between a male‐female pair of jaguars during and between potential mating periods. Ecologically, higher mutual information indicates that there is a stronger association of movement since more information about the movement of one jaguar is explained by the movement of the other.

I also note other methods that have been developed in recent years to model social interactions within the movement model as opposed to the post hoc measures of distance, cooccurrence potential, and correlation of movement (as measured by mutual information) (Scharf et al., 2016, 2018). Although several advantages exist in the use of such methods which rely on continuous‐time and nonparametric smoothing models, there remain important advantages of the use of semi‐parametric smoothing models (as are used in this paper) (Hamdy). An exploration of the properties of this proposed measure of localized mutual information has recently been conducted by proof and simulation under a variety of simple animal movement scenarios (Whetten, 2021).

### 2.5 Data: Taiama ecological station jaguar movement data

I add to the previous investigation of movement and social interaction of a collection of jaguars in the Taiamã Ecological Station, Pantanal, Brazil. The majority of jaguars examined in this project were fitted with Lotek GPS Iridium satellite collars and monitored for periods of 60 to 591 days (Morato et al., 2018). The movement of Jaguar 88 was monitored using a Lotek GPS GlobalStar satellite collar. The authors of the study have made it public and freely available at https://doi.org/10.1002/ecy.2379 and also at Dryad Digital Repository (https://doi.org/10.5061/dryad.2dh0223). In this project, I utilized data from the full monitoring periods on 7 jaguars from this region, and Table 1 presents the number of recordings and the length of the monitoring period. The finest temporal resolution of the data is on hourly intervals; however, there are frequent gaps in recordings where missing measurements may be present for 2 h to several days. It is important to disclaim that the authors of these data did not provide an estimated or empirically computed measure of error radius associated with each position. Instead, they have reported a dilution of precision of less than 10, which provides moderate to good levels of confidence in animal position. Dilution of precision refers to the quantification of error propagation in satellite navigation on the precision of estimated position (Langley, 1999). Previous work using these data product has also not incorporated the use of measurement error (Fontes et al., 2021; Morato et al., 2018, 2021). Animal movement data with an unreported measurement error are not ideal, but the aim of this project expands on previous analysis in an effort to provide further understanding of jaguar behavior and interaction. Their findings on jaguars in this region were accomplished by measuring and studying the cooccurrence and correlation between several pairs of jaguars. Using trajectories and association rule mining algorithms and a distance radii of 200m and 400m, they were able to estimate cooccurrence frequency and a single correlation metric for each jaguar pair (Fontes et al., 2021). Following the results section, I discuss the differences between this analysis and previous work which primarily pertain to the differences between cooccurrence frequency and cooccurrence potential and quantifying the correlation between pairs of animals.

**TABLE 1:** Monitoring statistics of Jaguars from the Taiamã Ecological Station

Jaguar local ID	Frequency	Monitoring period
12	2681	12/5/14–4/18/15
13	5040	12/7/14–8/24/15
18	2314	11/29/14–4/13/15
22	4709	9/11/14–5/21/15
41	4952	12/5/14–8/17/15
81	10,988	10/15/13–5/29/15
88	1296	10/9/13–4/20/14

The jaguars examined in this project were selected on the condition that they shared an overlapping monitoring period with at least one jaguar from the monitoring period with the highest activity monitoring period from December 2014 to the summer of 2015. The final and more detailed investigation of social interactions is performed for Jaguars 12, 13, 18, 41, and 81.

For all visualizations used in this work, I numerically transform to time in days from the earliest available date 10/9/13 for Jaguar 88. As such *t* = 0 is the first day recorded for Jaguar 88, and the final day on this scale is t=591 when the final measurement on Jaguar 13 is recorded, 08/24/15. This is particularly useful for monitoring periodicity and duration of events, since it is difficult to quickly understand the number of days or weeks between two dates.

## 3 RESULTS

**FIGURE 1:**
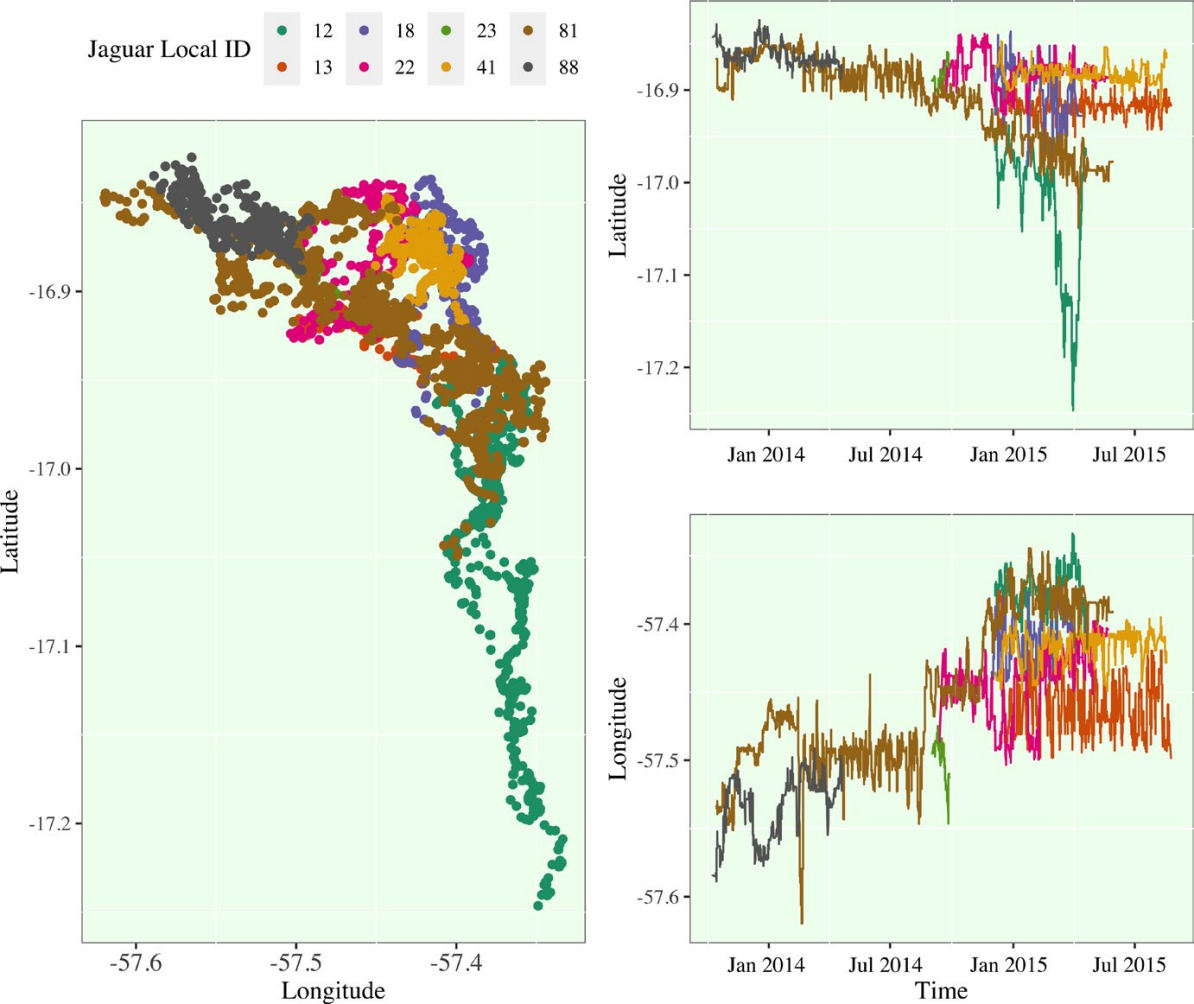
Visualization of Jaguar Movement in the Taiamã Ecological Station. (Left) The spatial distribution of GPS recordings is plotted and colored by Jaguar ID. (Right) The temporal change in each Jaguar's latitudinal and longitudinal position (Baptiste, 2017; Wickham, 2016). A terrain map of this region is provided in Figure S1

The primary challenge in mapping and analyzing relationships between Jaguars at the Taiama Ecological Station is the staggered time windows that each Jaguar is monitored coupled with the inconsistent temporal resolution of GPS readings. To reach our final selection of 7 jaguars, we removed two jaguars with less the 100 GPS recordings and two jaguars (Jaguar 91 and 92) that were monitored many months after the remaining jaguars (Jaguar 116 and 117). There are 3 females (Jaguars 12, 41, and 88) and 4 males (Jaguars 13, 18, 22, 81). I visualize the remaining 7 jaguars in Figure 1. Across the three plots provided, we can develop a short narrative of a few major movement characteristics. Within their respective time domains, most of the 7 jaguars have stable fluctuations in position within their territories (with some clear overlap in territories) (Eriksson et al., 2021; Fontes et al., 2021). However, Jaguar 81 (male, age = 4 years), the jaguar with the longest monitoring window, makes a significant territorial transition from residing in the same region as Jaguar 88 (female, age = 5 years) to the territory of Jaguar 12 (female, age = 4 years). There appears to be a period of interaction between Jaguar 12 and Jaguar 81, and then, Jaguar 12 makes a temporary but significant migration south for approximately 3 months before returning to the same region again as Jaguar 81. There are other male‐female interactions that not as easily discernible, and more investigation is clearly required.

**FIGURE 2:**
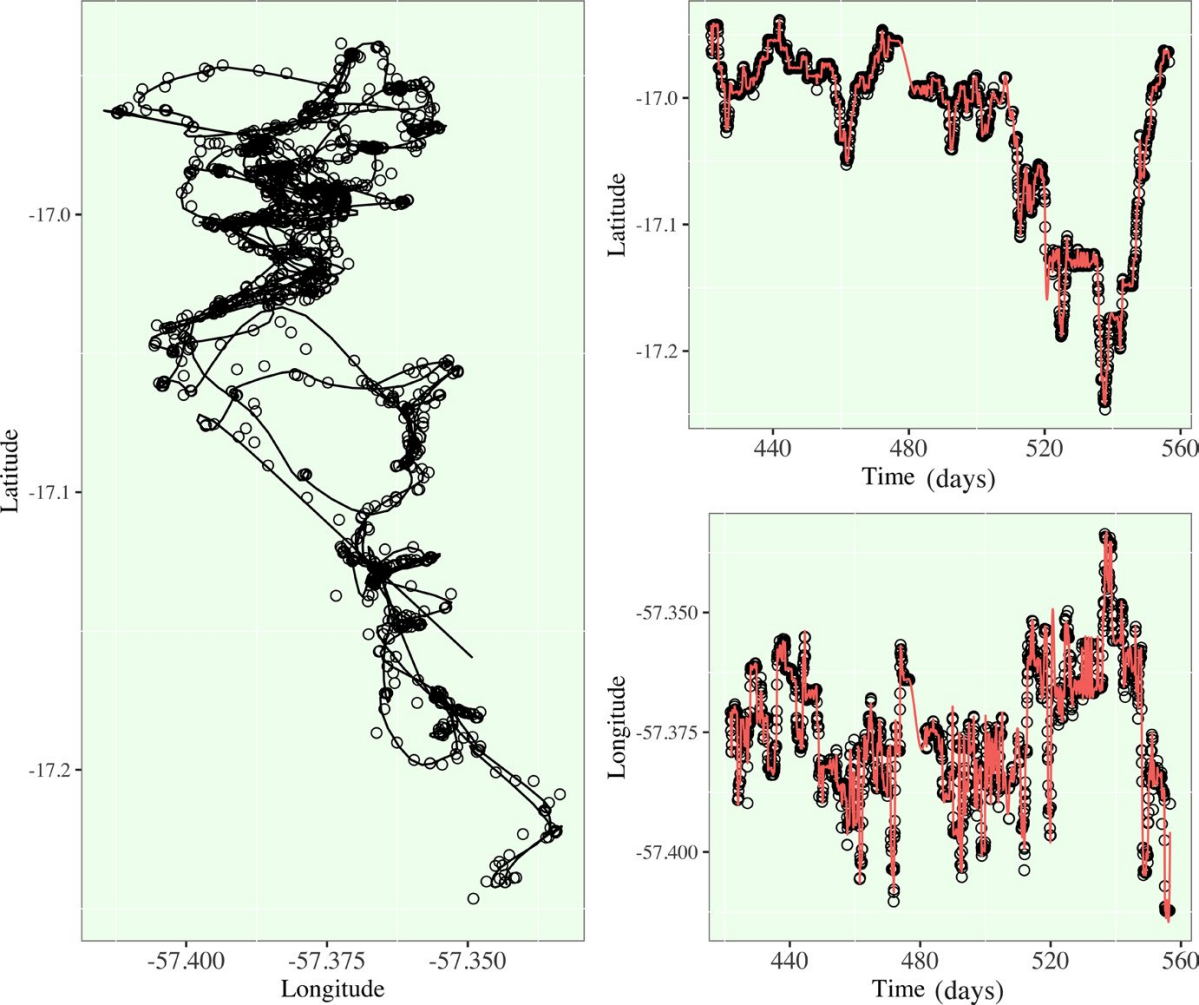
Smoothing Spline Model for Jaguar 12 (female, age = 4). (Left) The raw latitude‐by‐longitude position and spline model estimations are overlaid. (Right) The raw and smoothed components (latitude and longitude) are plotted with respect to time in days where *t* = 0 identifies the beginning of the study period in this region

**FIGURE 3:**
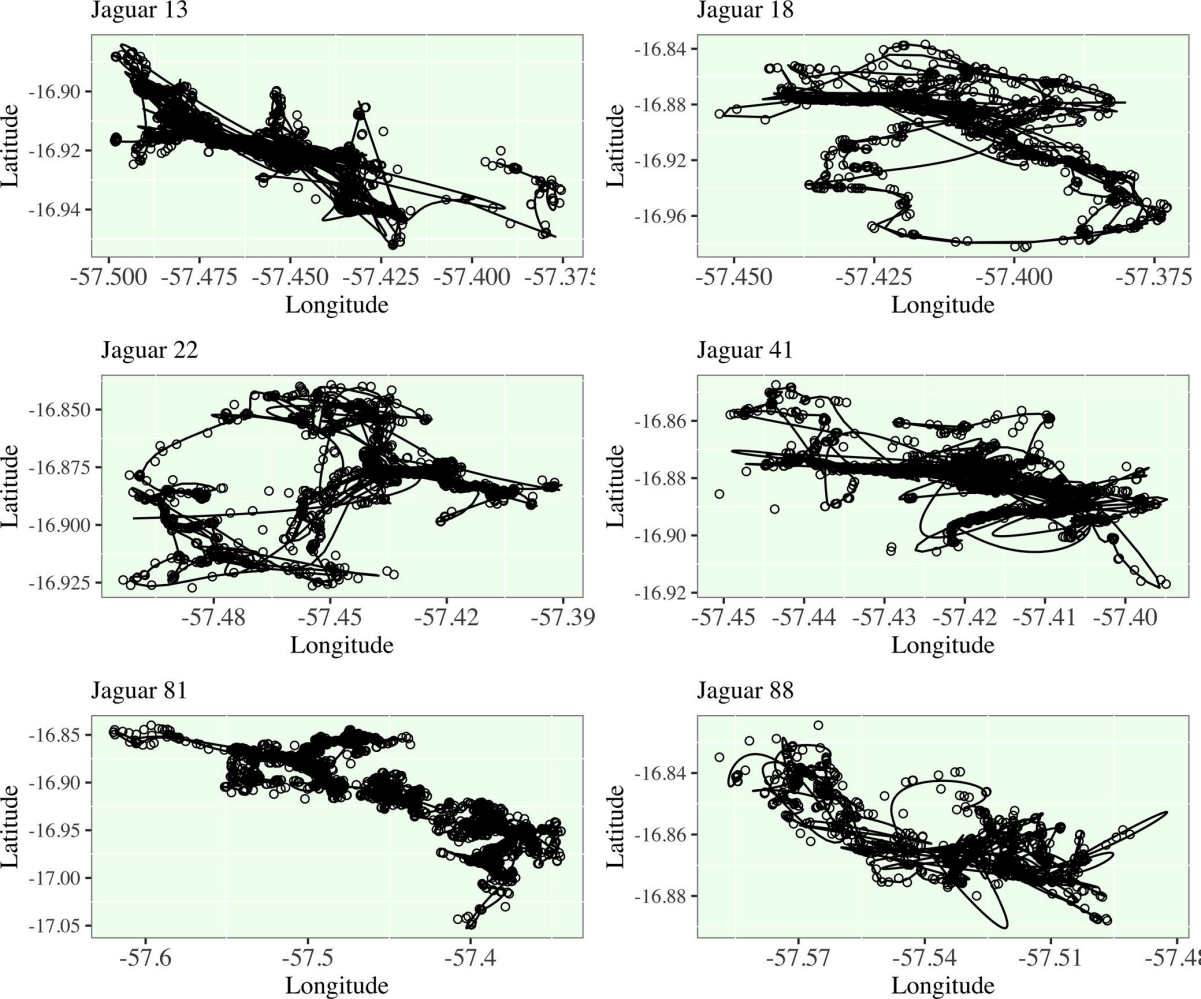
Smoothing Spline Model for Jaguars 13, 18, 22, 41, 81, and 88. For brevity, the decomposition of the spline models to latitude and longitude is not shown

In Figure 2, I present a detailed visualization of the smoothing of Jaguar 12's residential to migratory transition. As a can be seen visually, the fit of this spline model is exceptional and only a small selection of points are not well fit to the estimated path. This is an acknowledged sacrifice of information, in exchange for a number of benefits, primarily the refinement of the time resolution and consistency to a uniform time grid shared by all jaguars. In Figure 3, I present the smoothed spline models for the remaining seven jaguars. Further tuning of the model for Jaguar 41 and 88 should be considered as some raw locations are not well‐estimated, but Jaguars 13, 18, 22, and 81 have exceptionally well fit models. A well‐fit model loosely refers to a spline model that captures the raw movement path with reasonable accuracy from a visual inspection, and few positions are poorly estimated. The remaining jaguars have some points that the spline models did not fit as well under the general temporal density distribution procedure for knot placement documented in the methods section. There are some cases where it appears that the spline model “overshot” the path when an animal changed direction suddenly, or where there were a couple outlier points that the algorithm did not prioritize fitting. When optimizing a spline model overall minimization of the error is prioritized as opposed to local minimization of the error. Improvements to the models could be achieved by increasing knot densities in regions where it appears that the model is not fitting as well as other regions or deriving a localized spline modeling procedure that performs piecewise error minimization; these options are left to future work. We will use these models as is, since the deviations from the raw movement path are still limited, and most of movement profiles from these jaguars are well captured, meaning that the model is estimating a smoothed path through the majority of raw positions while avoiding overfitting to the exact positions in the raw data. All smoothing spline models have been smoothed to estimate behavior on a 1 h resolution.

**FIGURE 4:**
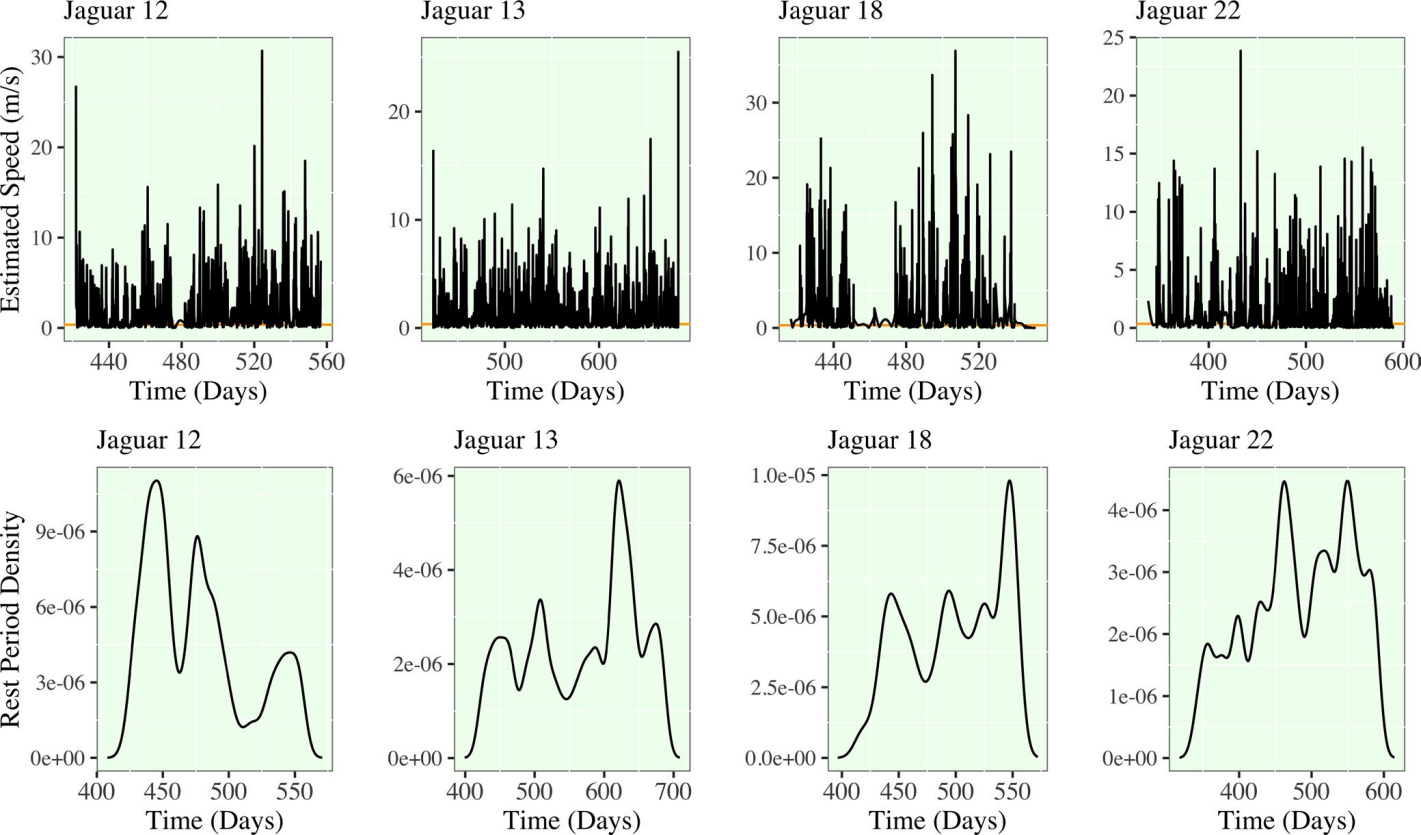
Spline model estimation of speed and rest period density for Jaguars 12,13,18,22. (Upper) A horizontal orange line is plotted at a speed of 0.25 m/s. (Lower) All hours in the spline model that are estimated to have speeds lower than this line are subsetted as a new vector to compute the density of rest periods. The selected bandwidth for estimation varies by jaguar and they range from approximately 4 to 12 days. As a result, a detected shift in the density of rest periods over time would indicate a shift to lower or higher density of rest periods occurring in a 4‐ to 12‐day window

**FIGURE 5:**
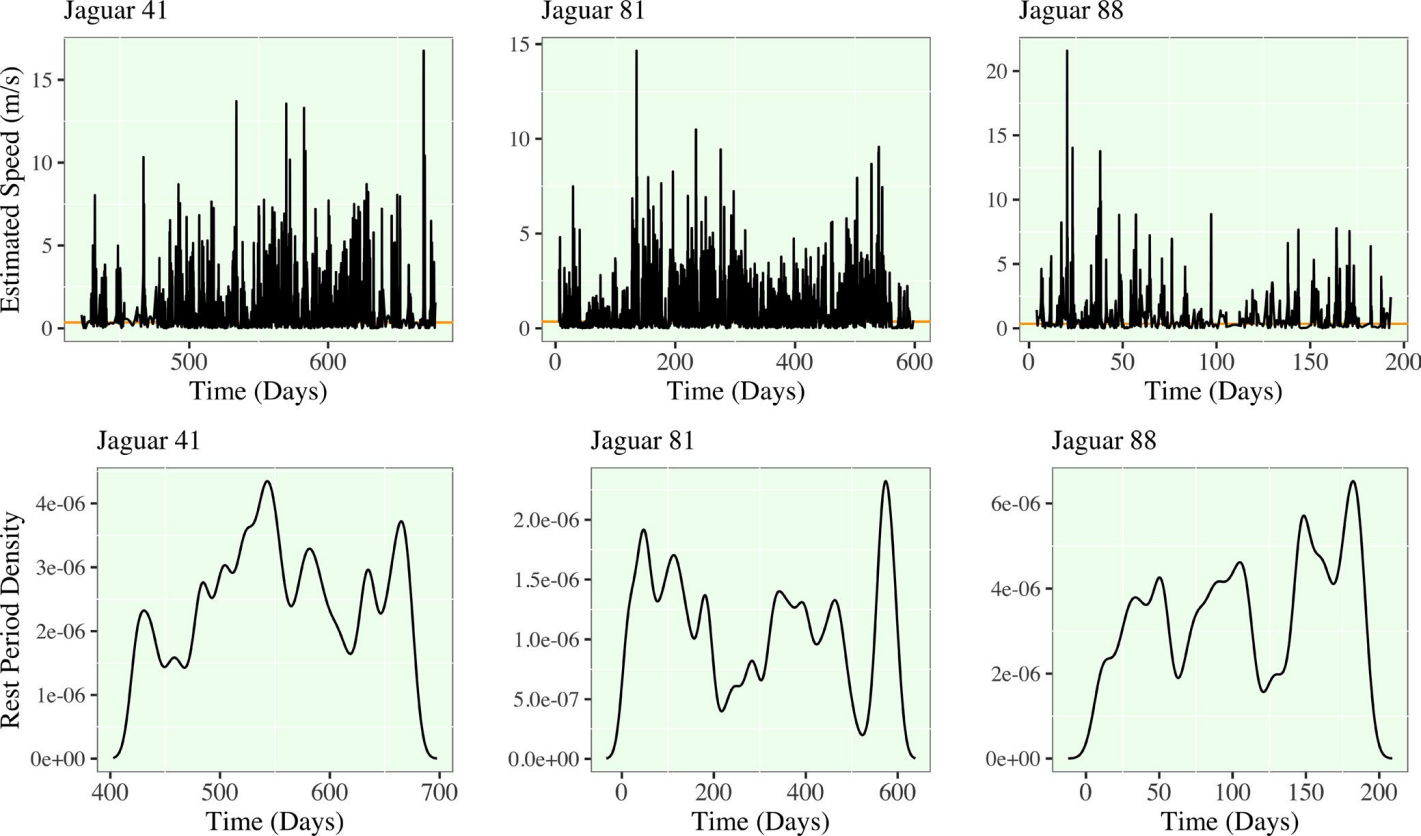
Spline model estimation of speed and rest period density for Jaguars 41, 81, and 88. Refer to the caption of Figure 4 for the interpretation

In Figures 4 and 5, I present the first derivative functions of the each jaguar's movement, as well as, the density of rest periods. Rest periods are defined (with some level of arbitration that is worthy of discussion) as times when the estimated speed of a given jaguar is less than 0.25 m/s. Any times where this condition is satisfied are found below the orange line. My working definition of a jaguar rest period is inherently a binary classification of movement, and the times that satisfy this condition are subsetted to derive rest‐period densities. I emphasize the substantial shift in the rest‐period density structure of Jaguar 12. Jaguar 12 in the first half of her tracked time domain has higher rest period density, meaning that she is estimated to have more rest periods or periods of slower movement. In the latter half, her rest period density drastically drops to below a third of previous levels. No other jaguars show this trends as drastically; Jaguar 81 has a drop in rest period density during the migratory period prior to entering the initial territory of Jaguar 12. In all of the remaining density plots, however, there is an apparent cyclic nature to rest period density that is approximately weekly to bi‐weekly for most jaguars.

**FIGURE 6:**
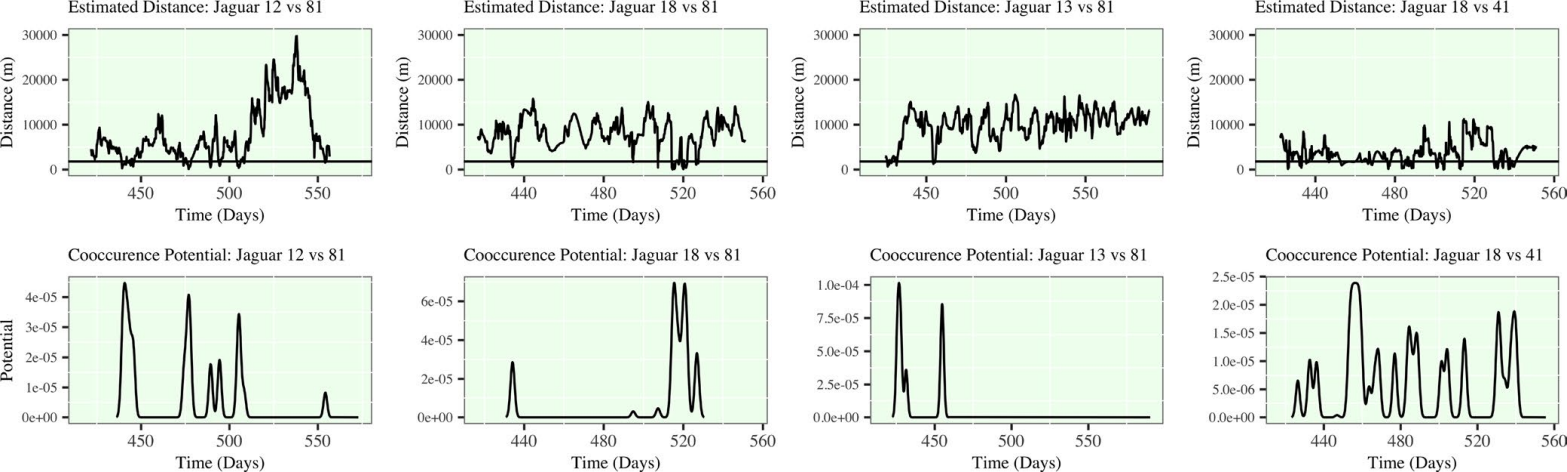
(Upper) Distance plots for Jaguars 12–81, 18–81, 13–81, and 18–41. Distance is derived from pairs of smoothed spline models. A horizontal line at Distance = 1800 m is placed to mark the defined threshold of cooccurrence. (Lower) All times that distance between a pairs of jaguars are subsetted to derive the density of times where jaguars fall within this threshold. The spacing and duration of close proximity is accentuated and this measure of cooccurrence provides easy access to measures of duration and frequency of cooccurrence or gaps in cooccurrence

In Figure 6, I present the pairwise distance relationships between several jaguar pairs, and their respective cooccurrence potential measures (Hijmans, 2019). The four selected pairs are chosen deliberately as many jaguars had zero or near zero cooccurrence potential. The male–female pairs are Jaguar 12 and 81 and Jaguar 18 and 41, and the male–male pairs are Jaguar 18 and 81 and Jaguar 13 and 81. In the distance function plots, which are all identically scaled on the vertical axis from 0 to 30,000 m, we note the significant differences in distance functions across all chosen pairs. For Jaguar 12 and 81, there is a first encounter with the highest cooccurrence potential, and then, there is an extended period of zero cooccurrence potential. Following this hiatus, there is an extended period of regularly occurring bursts of high cooccurrence potential, which is then followed by the long migration of Jaguar 12 away from Jaguar 18. At the end of their shared time domain Jaguar 12 returns and there is a short period of moderate cooccurrence potential that is evidence of some final return to territory sharing before we lose sight of their movement. The other male‐female pair (Jaguars 18 and 41) on the other hand, has regular intervals of high cooccurrence, but we note that in a similar seasonal time window (at approximately Day 500) Jaguar 41 distances herself from Jaguar 18, but at a much lower magnitude than Jaguar 12.

For the male–male pairs, Jaguars 13 and 81 only have high cooccurrence for a small time‐window, while Jaguar 81 is still migrating to new territory. Jaguar 18 (M) and Jaguar 81's (M) relationship is particularly interesting as there is an early period of high cooccurrence, and then, during the period of high cooccurrence between Jaguar 12 (F) and 81 (M), there is a hiatus in their cooccurrence. High cooccurrence between these two males is then resumed once Jaguar 12 (F) leaves the territory and they move within short distances of each other for an extended period, which ends before the return of Jaguar 12.

**FIGURE 7:**
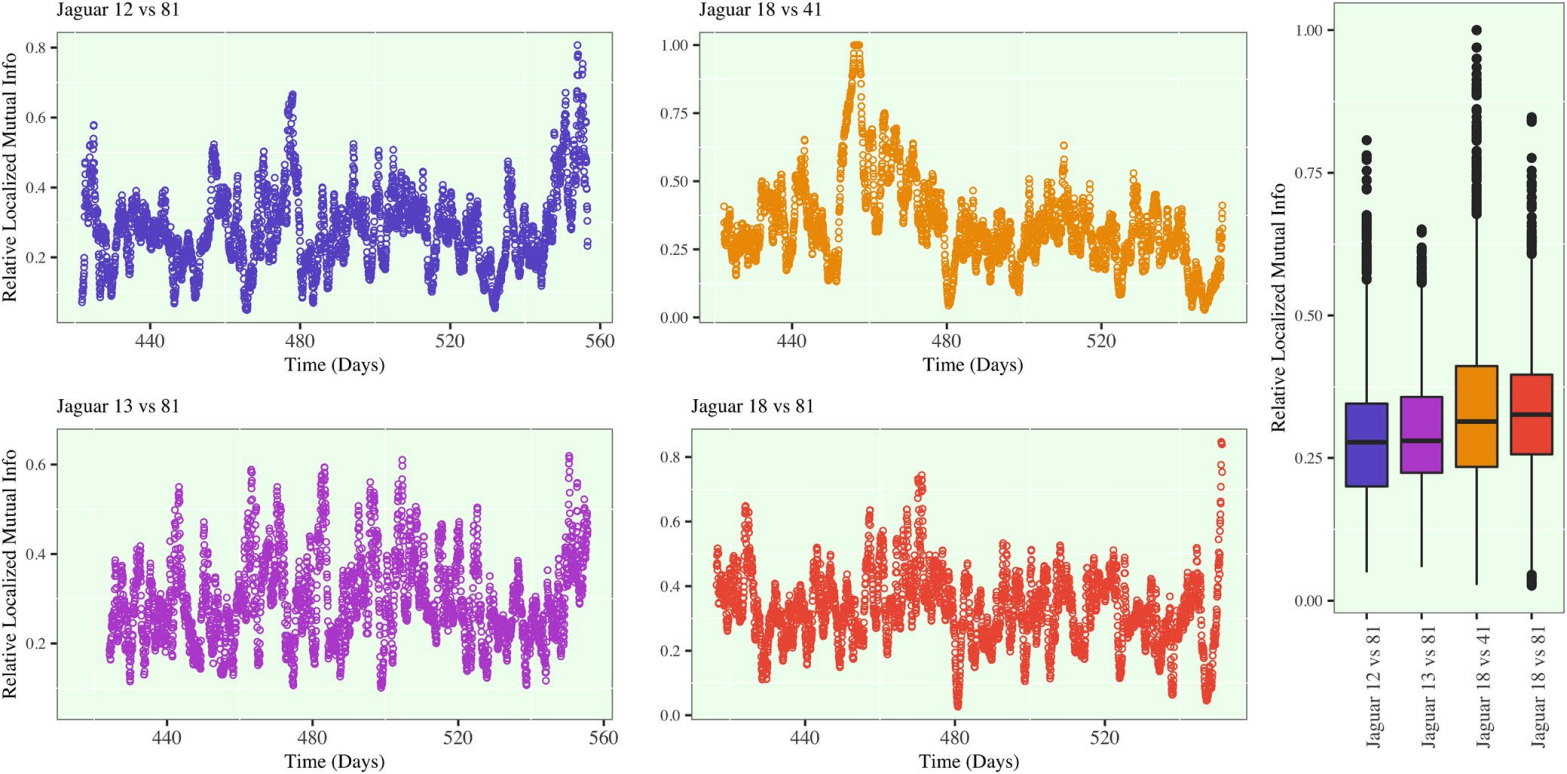
Localized mutual information plots. (Left) The localized mutual information with a bandwidth of *λ* =48 h for each time point in the refined time grid is plotted by each pair of Jaguars. The *y*‐axis is scaled by the maximum localized mutual information at each time point, and as a result, the range of the *y*‐axis is from 0.00 to 1.00. As a result, the scaled localized mutual information can be handle similarly to a measure of correlation, where 0.00 denotes no correlation between the movements and 1.00 defines a perfect unity in movement. (Right) The overall spread of localized mutual information measures across the time grid is summarized using boxplots

The localized mutual information profiles for the same four pairs jaguars are shown in Figure 7 using a bandwidth of *λ* = 48 h. This bandwidth identifies that the measure of localized mutual information is computed for a 4‐day period centered on a given time. For Jaguars 12 (F) and 81 (M), there is a cyclical spike in the strength of association (i.e., local mutual information) immediately prior to and during most of the periods of high cooccurrence potential. The times of strongest association in movement occur during the second and longest period of high cooccurrence from approximately Day 475 to Day 510, and when Jaguar 12 returns at the end of the study period. On the other hand, Jaguars 18 and 41, although regularly experiencing period of high cooccurrence, do not show a similar associative trends. Their movement has the strongest association early in the study period, and then, it gradually declines in the following weeks.

For the male–male pairs of jaguars, there are repeated periods of high mutual association that do not show clear trends with cooccurrence potential. For Jaguars 13 (M) and 18 (M), there is a drop in the strength of association in their movement in the final weeks of the study and this is when these two jaguars are consistently the furthest apart. Interestingly, Jaguars 18 and 81 (male–male pair) have the strongest association in their movement at a similar time to the peak in association between Jaguars 12 and 81 (female–male pair). The female–male pair have a peak in association at Day 478, and the male–male pair have a peak in association at Day 471. These two associations are characterized by a zero‐level cooccurrence between the male pairs of jaguars, and increasing cooccurrence potential between the female–male pair.

In order to further investigate the cause of these spikes and drops in mutual information, it becomes highly instructive to summarize the pairwise distance and localized mutual information for these pairs of jaguars in addition to the pair of Jaguars 18 (M) and 41 (F). This is shown in Table 2. Between Days 476 to 479, Jaguars 12 (F) and 81 (M) are in close proximity with an estimated median hourly distance of 1212m apart and a minimum estimated distance of 118.4m apart. The spike in their correlatory movement peaks in this window marks that their relationship in this time is characterized by periods of direct interaction. Jaguars 18 (M) and 81 (M) during their peak in movement association in the time window from Day 469 to Day 471 is not direct as the minimum estimated distance between these jaguars is 7069m. However, in the time window from Day 455 to Day 477 (which overlaps this time), Jaguars 18 (M) and 41 (F) have an estimated median distance of 2071m and a minimum distance of 156 m with extended periods of high cooccurrence potential. *What is especially interesting about these observations is that the peak in association between the two male jaguars is that it characterized by a periods when both male jaguars have close interaction with females*. The comparison of localized mutual information plots for Jaguar's 12, 18, 41, and 81, provides a clear characterization of interaction on a local male and female jaguar behaviors.

**TABLE 2:** Peak association in between Jaguar Pairs

Jaguar pair	Local time window	Metric	Min	Q1	Q2	Q3	Max
12 (F) vs. 18 (M)	Day 476–479	Distance	188.4	591.1	1212.0	2299.0	2874.1
		Mutual Information	0.385	0.447	0.499	0.612	0.667
18 (M) vs. 81 (M)	Day 469–471	Distance	7069.5	9174.9	10,641.1	11,562.3	12,537.0
		Mutual Information	0.316	0.416	0.519	0.637	0.744
18 (M) vs. 41 (F)	Day 455–477	Distance	156.0	1736.0	2071.2	2922.6	4039.1
		Mutual Information	0.266	0.406	0.515	0.634	0.999

Note

Distance and localized mutual information are summarized by quantiles for pairs 12 vs. 81, 18 vs. 81, and 18 vs. 41 for a time window of interest surrounding a peak in association of movement as measured by localized mutual information.

## 4 DISCUSSION

The apparent complexity of jaguar movement and interaction in the Taiamã Ecological Station is driven by the high density of jaguars (Fontes et al., 2021; Morato et al., 2016). Monitoring the complex fine‐scale movement of multiple animal with shifts in territorial and social nature differs from previous examinations of animal movement using smoothing spline models (Buderman et al., 2016; Hefley et al., 2017; Henning et al., 2017; Hooten & Johnson, 2016). This work provides a preliminary strategies for monitoring movement, behavior, social interactions, and the strength of association between animal movement, all of which are best explored on a refined and unified time grid smoothed using spline models.

The Taiamã Ecological Station is a crucial conservation region for jaguars, and it is the region with the largest known density of jaguars, and further, this region provides insights into the needs of an ecosystem to sustain a large volume of neotropical apex predators (Cavalcanti et al., 2012; Kantek & Onuma, 2013; Morato et al., 2016). Recent work has shown that the size of this conservation region is insufficient to protect this specific feline population. The study of space‐use and animal interaction is a crucial step to assessing the conservation needs for this species (Cullen et al., 2013; Sollmann et al., 2008).

As the objective of this work is comparative to the recent work on pairwise jaguar interactions, I compare the primary differences and potential advantages over Fontes et al.

### 4.1 Cooccurrence potential vs. cooccurrence frequency

Although measurement error is not considered in either analysis, it is crucial to acknowledge error in position and choose a metric for examining distance between animals that accounts for movements that may occur between known positions. Cooccurrence frequency is defined in previous work on a very close proximity of 200m and 400m, which can be advantageous in the sense that jaguars within this range are almost surely aware of each other. However, simply counting the instances of cooccurrence in this way does not provide a tool for measuring and visualizing periods where cooccurrences are realized in high or low densities. It is clear that a period with a high density of cooccurrences is more likely to contain interaction since there are more opportunities for an interaction to take place. Given that the raw recordings are at best defined on a 1‐hour resolution, it is evident that even over dense or difficult terrain, jaguars have the potential to cover a distance many magnitudes farther than 400m. Additionally, loud mating calls are used by the species to attract mates far beyond this range, and scent and scrape markings are other methods of communication by this largely solitary predator (Palomares et al., 2018). Cooccurrence potential in this work is a simple extension of cooccurrence frequency where a larger distance is used for measuring frequency, and the densities of these frequencies are used to derive a probability density function that identifies time windows where there is a greater probability of an interaction. The visualization of cooccurrence in this way provides a convenient tool for examining complex patterns in cooccurrence between animals or differences between pairs of animals.

### 4.2 Localized mutual information functions vs. trajectory and association rule mining correlation metric

The derivation of a single correlation coefficient as laid out in Fontes et al. it attractive in its simplicity and use of interpretable association rules. However, as is evident in the relationship between Jaguar 12 and Jaguar 81, shifts in behavior states over time result in shifts in the association of movement. This is apparent for most pairs of jaguars. There is always some level of associations between animals of the same species in the same local ecosystem, even if there is no direct interaction. As an example, as shown in the state matrices in Fontes et al. (2021), there are overlapping times of day where Jaguars are resting or in other transitory states. It follows that some mutual information between jaguars is evident as movements in the region may be connected through animal gender, daylight, and shifts in weather or climate. In the localized mutual information functions derived in this work, spikes or periods of relatively higher correlation denote stronger relationships between a pair of jaguars. More simply, local mutual information functions can be thought of as a time‐dependent measure of correlation or association of animal movement. The ability for this method to captures association of movement regardless of direct proximity is a critical advantage in adapting correlation analysis of animal movement beyond cooccurrence studies. This is most clearly illustrated in the ability of the proposed localized mutual information measure to detect a spike in similarity of movement between two males (Jaguars 18 and 81) when they are both interacting or in close proximity of a female. It is only after this spike that they move closer to each other and observe a spike in cooccurrence potential.

It is important to note that the proposed LMI measure and movement associations driven by scent and scrape marking patterns should be explored further via simulation studies. The integration of GPS tracking data and geographical distribution of scent and scrape marking patterns would permit for the testing of the influence of scent and scrape marking behavior on animal movement (Towns et al., 2017). This would provide crucial insight since scent and scrape marking data would represent the true population of jaguars of a region which may assist in explaining behaviors of observed jaguars in the presence of unobserved jaguars. The spike in the Jaguar 18 and 81's cooccurrence potential following provides evidence of increased male‐male interaction only once females have distanced themselves from each respective male.

### 4.3 Extension to higher‐order interactions

Although not shown in this work, the methods implemented have the ability to be extended to monitor three way interaction. Cooccurrence potential for any given jaguar, in relation to two or more other jaguars, would be the density function of time recordings on the refined time grid where any jaguar is within a set radius (such as 1800 m). The localized mutual information measure could be readily adapted to measure partial mutual information (Darudi et al., 2013), where the association between two jaguars is measured while controlling for another jaguar. In both this work and Fontes et al., the interpretations are based solely on observed individuals, and there are still challenges present in interpreting interactions detecting between pairs of jaguars when there are likely other interactions with unobserved individuals.

As mentioned briefly above, jaguar social interaction, although primarily characterized by direct (or close‐proximity) interaction, is not the only form of social interaction that exists and should be detectable. Like many apex predators, territorial marking, is a common form of passive communication. Jaguars may deliberately avoid or follow these routes which should be characterized by higher associations between animals. Young male have a tendency to be nomadic and older jaguars tend to have established territory with minimal overlap (where overlap is typically shared with females in the region). Female jaguars behavior is also generally characterized by a temporary associations with a male, and then, they avoid male interactions when caring for cubs (Azevedo & Murray, 2007; Cavalcanti & Gese, 2009; Conde et al., 2010; Harmsen et al., 2010; Towns et al., 2017).

All of these characteristics of jaguar movement and interaction are detectable in this analysis. Jaguar 12 (female; age = 4) and Jaguar 81 are detected to have strong but temporary associations which increases in frequency as time progresses, and then, there is a rapid distancing between the pair and the association in their movement drops for over two months. The ability to detect an increase in frequency in high cooccurrence is visually inconclusive without the use of cooccurrence potential plots. Finally, their association and cooccurrence potential increase at the end of the study as she returns to her baseline territory at the beginning of the study. Jaguar 12's resting behavior also shows distinct shifts from the period of high cooccurrence potential with Jaguar 81 to the farthest point in her migration south. It is suspect that Jaguar 12's sudden drop in rest period densities suggests a shift between mating and cub rearing movement behaviors where she is depended on to make successful hunts to provide for her young. Females are generally considered to have smaller home ranges, but the seasonal shifts in this Jaguar 12's behavior for months of this year show evidence that some females have multiple or shifting home ranges during mating and cub‐raising periods (Morato et al., 2016). That fact that some female jaguars make longer temporary migrations proceeding interactions with male should be considered when defining an appropriate conservation region for the species since the time spent away from males is a critical time for survival of the next generation of cubs.

The nomadic behavior of Jaguar 81, which is recorded at a fine scale for almost two years, provides particular insights regarding male–male relationships between established and nomadic male interactions. Jaguars 13 and 81 only seem to interact for a brief time in passing, and Jaguar 81 continues to move past Jaguar 13's territory. However, Jaguars 18 and 81 show evidence of coexisting in a similar region with distinct shifts in behavior. Jaguars 18 and 81 have the strong associations in movement in the presence of a local female. Jaguar 18 keeps at a farther distance from Jaguar 81 once high cooccurrence between Jaguars 12 and 81 begin, and Jaguar 18 is not shown to near Jaguar 81 until Jaguar 12 has initiated a prompt departure from the region.

The migrations of Jaguar 12 and 81 provide evidence that interacting with high cooccurrence potential in regions of high population density utilize expansive regions of land (upwards of 30km) (Morato et al., 2016). This is critical to understand as conservation efforts demand estimations of the required conservation area for endangered species (Cavalcanti et al., 2012; Kantek & Onuma, 2013). With any region of higher jaguar density, this work confirms that increasing conservation land for jaguar's will only aid their ability to coexist in higher abundance (Sollmann et al., 2008), since longer migration's (greater than 30 km) of a terrestrial predator could easily span outside of protected areas with a radius of less than 60 km. As the movement of all jaguars in this region is not observed, it would be hypothesized and left to future work to examine how often migrations of this level take place in regions with high densities of apex neotropical predators.

Smooth spline modeling of jaguar movement, as demonstrated in this study, is not without some caveats that should demand further attention in future work. As mentioned earlier, smoothing of paths requires some sacrifice of the exactness of position, and some particular movements are more difficult to catch than others. For animal telemetry, spline models are subject to over‐ and under‐fitting challenges which can be observed in Figure 3. Some paths are clearly more variable than the smoothed model suggests, and depending on the density of time measurements in some region, the model may tend to overshoot or undershoot a sharp change in direction. As in recent developments in standard continuous time models, there are opportunities to improve the fit of the model by accounting for geographic features/barriers, social encounters, atmospheric conditions, etc. (Togunov et al., 2021). Random walk schematics have shown great potential improving the modeling of animal movement, and these methods should be adapted to the FDA paradigm.

In this analysis, there is no accounting of measurement error, which is a significant element of most animal telemetry data. The data used in this work did not publicly provide measurement error to pair with GPS point estimates of position. As mentioned previously, some recent work has provided possible methods for accounting for measurement error in spline models, and these should be adaptable to many applications in animal movement.

The use of information theory in animal telemetry is sparse, but this work demonstrates the value of adapting measures of entropy and mutual information to animal telemetry. The derived measure of localized mutual information verifies that although the distance between jaguars has a tendency to yield higher associations in their movement, this is not uniformly true and there are strong associative movements between male–male and male–female pairs that can occur far beyond the cooccurrence potential threshold that I have defined in this work.

In overview, the approach used in this work effectively handles the challenges of spatial and temporal density, modeling continuity and differentiability of spatial movement, and multivariate characterization of animal behavior. To elaborate on the latter, the spline models that I construct in this work retain information about animal position and rate of change of position while refining the movement uniformly with other animals, which ultimately allows for a unique and visual‐friendly characterization of shifts in interaction and social behavior.

I commend past work in the study and modeling of animal telemetry, social interaction monitoring, and I encourage further work in modeling of these complex processes and relationships.

## CONFLICT OF INTEREST

The author declares no conflict of interest.

## AUTHOR CONTRIBUTION

**Andrew B. Whetten:** Conceptualization (equal); Data curation (equal); Formal analysis (equal); Investigation (equal); Methodology (equal); Project administration (equal); Software (equal); Validation (equal); Visualization (equal); Writing‐original draft (equal); Writing‐review & editing (equal).

## Supporting information

Appendix S1

Supplementary Material

## Data Availability

Jaguar movement database: a GPS‐based movement dataset of an apex predator in the Neotropics (Langley, 1999; Morato et al., 2021). The authors of the study own the data set and made it public and freely available at the Dryad Digital Repository with the following DOI accession number: https://doi.org/10.1002/ecy.2379. It can also be accessed via the following link: https://doi.org/10.5061/dryad.2dh0223. The data are also available on Movebank at https://doi.org/10.5441/001/1.3c4fv0m4.
