# Supplementary figures and images for: Smoothing splines of apex predator movement: Functional modeling strategies for exploring animal behavior and social interactions
Source: Ecol Evol. 2021 Dec 9;11(24):17786–800. doi: 10.1002/ece3.8294 (PMC8717279; doi:10.1002/ece3.8294)

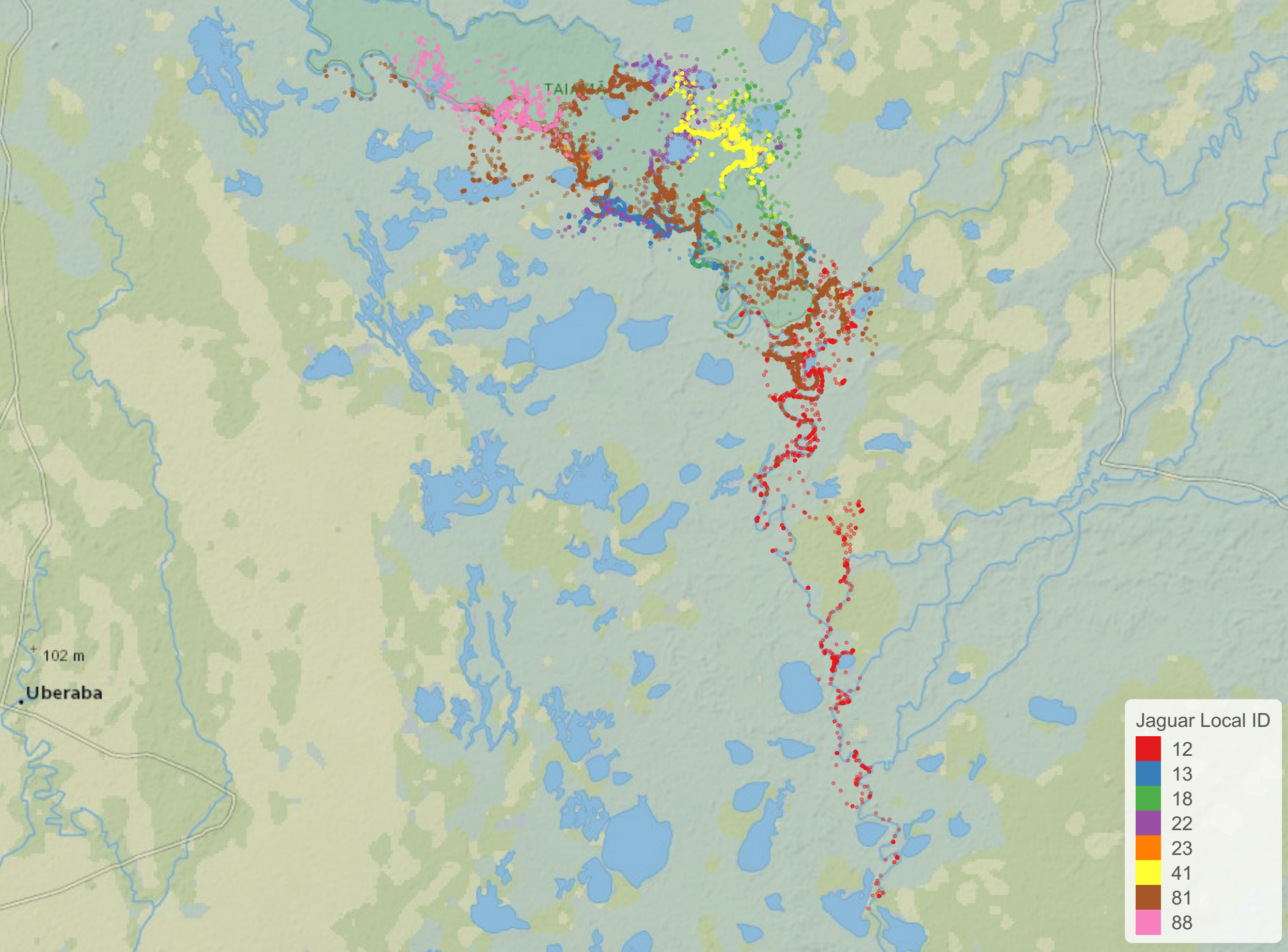

Supplement: Supplementary file 1 — Appendix S1 [file ECE3-11-17786-s001.pdf]
