# Supplementary material for: Smoothing splines of apex predator movement: Functional modeling strategies for exploring animal behavior and social interactions
Source: Ecol Evol. 2021 Dec 9;11(24):17786–800. doi: 10.1002/ece3.8294 (PMC8717279; doi:10.1002/ece3.8294)
Supplement: Supplementary file 2 — Supplementary Material [file ECE3-11-17786-s002.docx]

Appendix 1

Figure S1: Terrain image base layer of jaguar spatial distribution in the Taiamã Ecological Station. It is important to note the movement around waterways. A well fit smoothed spline model should generally avoid fitting paths directly through natural barriers that restrict jaguar movement in areas with a generous density of measurements in an area, but future work should be done to ensure that a fitted spline model avoids drawing an unrealistic path in areas that have sparse data.
